# Effect of Dealcoholized Muscadine Wine on the Development of Spontaneous Colitis and Gut Microbiome in IL-10^−/−^ Mice

**DOI:** 10.3390/nu17142327

**Published:** 2025-07-16

**Authors:** Hao Li, Liwei Gu

**Affiliations:** Food Science and Human Nutrition Department, Institute of Food and Agriculture Sciences, University of Florida, Gainesville, FL 32611, USA

**Keywords:** IL-10^−/−^ mice, dealcoholized muscadine wine, colitis, gut microbiome, diet-gene interactions

## Abstract

**Background/Objectives**: Colitis is a chronic condition affecting millions worldwide. Purple muscadine wine polyphenols have a unique composition and possible disease-preventive properties. This study aims to determine how dealcoholized muscadine wine (DMW) affects the development of colitis and gut microbiome in IL-10^−/−^ mice, compared to wild types (WT). **Methods**: Six-week-old male IL-10^−/−^ and WT C57BL/6 mice were fed either a DMW-supplemented diet (4.8% *v*/*w*) or a control diet based on AIN-93M for 154 days. Colitis severity was evaluated by disease activity, intestinal permeability, gene expression of cytokines and tight junction proteins in the colon, and inflammatory cytokines in the serum. Fecal samples were collected for gut microbiome profiling via 16S rRNA gene sequencing. **Results**: DMW contained predominantly anthocyanins and a significant amount of ellagic acid. IL-10^−/−^ mice developed mild colitis as indicated by the disease activity index. DMW × gene interactions decreased intestinal permeability, colonic mRNA levels of IL-1β, and serum TNF-α in the IL-10^−/−^ mice. DMW suppressed the colonic mRNA levels of IL-6, enhanced the gene expression of ZO-1, but did not influence the mRNA level of TNF-α or occludin. While DMW did not alter α-diversity of the gut microbiome, it significantly influenced β-diversity in the WT mice. DMW significantly reduced the relative abundances of *Akkermansia* in the IL-10^−/−^ and WT mice. DMW and DMW×gene interaction decreased the relative abundance of *Parasutterella* only in IL-10^−/−^ mice. **Conclusions**: These results suggested that polyphenols from DMW interacted with genes to moderately alleviate the development of colitis in IL-10^−/−^ mice and could be a useful dietary strategy for IBD prevention.

## 1. Introduction

Inflammatory bowel diseases (IBD), consisting of Crohn’s disease and ulcerative colitis, are a group of chronic and recurring inflammatory conditions in the colon and intestine. The prevalence of IBD grows steadily in early industrialized countries, while its incidence increases in newly industrialized regions since 2000 [1]. Common symptoms of IBD include abdominal pain, diarrhea, fever, fatigue, and blood in the stool. IBD increases the risk of colorectal cancer by 2–3 fold [2].

Many studies have suggested that polyphenols are effective in preventing the development and managing the symptoms of IBD. For example, hydroxytyrosol inhibited the TLR4/NF-κB signaling pathway, reduced colon expression of IL-6 and TNF-α, and increased the abundance of *Bacteroidota* in mice with DSS-induced acute colitis [3]. Resveratrol decreased the gene expression of IL-1β, TNF-α, and IL-6, but increased the expression of IL-10 in DSS-treated mice by inhibiting the JAK2/STAT3 pathway [4]. Polyphenol ligustroside preserved the structural integrity of the colon tissue and affected both the α- and β-diversities of gut microbiome, compared to DSS-treated colitis mice [5].

Muscadine grapes (*Vitis rotundifolia*) originate from the south-central and southeastern United States. Muscadine grapes or wines contain six anthocyanins, all in the form of 3,5-diglucosides. In contrast, common grapes contain primarily anthocyanins in 3-glucoside forms. They also contain other polyphenols, including ellagitannins, ellagic acid, myricetin, quercetin, epicatechin, and catechin, but with a low content of resveratrol [6]. High levels of ellagitannins and ellagic acid are also unique to muscadine grapes [7]. Muscadine grapes are characterized by thick, tough skin and large seeds. Partially due to the lack of seedless cultivars, over 90% of muscadine grapes are made into wine.

It was reported that muscadine grape extracts decreased the superoxide level in neutrophils of rats treated with phorbol myristate acetate. The levels of IL-1β, IL-6 and TNF-α were reduced in peripheral blood mononuclear cells stimulated by lipopolysaccharides. Muscadine grape skin extracts were also found to mitigate the paw edema in carrageenan-stimulated rats [8]. In addition, muscadine skin or seed extracts prohibited edema and decreased the myeloperoxidase activity in mice ears irritated by tetradecanoylphorbol acetate [9]. Muscadine wine polyphenols were shown to prevent weight loss and colon shortening, downregulate the NF-κB pathway and secretion of IL-1β, IL-6, and TNF-α in the colon of mice with DSS-induced colitis [6].

Many studies have demonstrated the effect of polyphenols on chemically induced colitis in mice. However, there are few studies investigating the effect of polyphenols on the colitis developed in genetically susceptible mice. IL-10^−/−^ mice have been widely used to investigate IBD because they develop colitis spontaneously [10]. After the onset of colitis, IL-10^−/−^ mice have a higher secretion of inflammatory cytokines than wild-type mice, including TNF-α, IL-1β, IL-6, and IFN-γ [11]. Previous studies demonstrated that muscadine wine polyphenols restored the gut dysbiosis in DSS-treated mice [12]. However, it is unknown whether muscadine wine has similar effects in IL-10^−/−^ mice. Nutrigenomics and nutrigenetics reveal the complex and bidirectional interactions between diet and genes and a vital role of such interactions in health and disease. Diets influence gene expression via epigenetic modification, transcription factor activation, and microRNA regulation. Genetic differences, such as single-nucleotide polymorphisms, enzyme function variability, and taste preference, affect how individuals metabolize and respond to nutrients [13]. Most of the previous research focused on the diet, while the interaction between diet and genes was ignored. Therefore, this study aimed to test the hypothesis that a diet supplemented with DMW interacts with genes to alleviate IBD in IL-10^−/−^ mice. Dealcoholized wine has been extensively used to study the nutritional properties of wine polyphenols in humans and rodents without the interference of alcohol. The non-alcoholic wine is also gaining popularity among consumers in recent years [14].

## 2. Materials and Methods

### 2.1. Materials

Muscadine wine (Lakeridge Winery, Clermont, FL, USA) was condensed to 10% of the original volume under partial vacuum to prepare dealcoholized muscadine wine. Standards of phytochemicals (anthocyanins, ellagic acid, myricetin, quercetin, and kaempferol) and FITC-dextran (4 KD) were provided by MilliporeSigma, Inc. (Burlington, MA, USA).

### 2.2. HPLC Analysis of Dealcoholized Muscadine Wine

To quantify anthocyanins, DMW was dispersed in methanol: water: formic acid (50:49:1, *v*/*v*/*v*). The filtered solution was injected onto an HPLC with ZORBAX C18 Column (5 µm, 4.6 × 250 mm, Agilent Technologies, Santa Clara, CA, USA). The mobile phase consisted of 5% formic acid (phase A) and 100% methanol (phase B). The linear gradient was 0–10 min, 5–15% B; 10–30 min, 15–30% B; 30–45 min, 30% B; 47 min, 70% B. The flow rate and detection wavelength were 0.8 mL/min and 520 nm, respectively. DMW was hydrolyzed before non-anthocyanins were analyzed on HPLC. One mL of DMW was mixed with 3 mL of 50% methanol and 0.45 mL of 37% (*w*/*w*) HCl. The mixture was placed in a water bath at 90 °C for 80 min. After that, the final volume of the mixture was adjusted to 5 mL with 50% methanol. The diluted and filtered solution was injected into an Agilent ZORBAX C18 Column (5 µm, 4.6 × 250 mm). Mobile phases included 0.5% formic acid in water (phase A) and acetonitrile (phase B). The linear gradient was 0–5 min, 10–30% B; 10 min, 40% B; 20 min, 50% B; 23 min, 70% B; 25 min, 10% B. The flow rate and detection wavelength were 1.0 mL/min and 360 nm, respectively.

### 2.3. Proximate Analysis of DMW and Diet Formulation

The total fiber and total protein in DMW were analyzed according to AOAC 991.43 and AOAC 991.20.I, respectively, at Mérieux NutriScience Inc. (Gainesville, FL, USA). Ash was analyzed in a furnace based on AOAC 942.15. Moisture was analyzed after DMW was dried in an oven at 105 °C for 3 h. Fat content in DMW was assessed using the chloroform-methanol method [15]. Sugar content was assessed on an Agilent HPLC with a refractive index detector. Sugars were separated on a Luna NH2 column (5 µm, 250 × 4.6 mm, Phenomenex, Torrance, CA, USA). The column was eluted with 75% aqueous acetonitrile at a flow rate of 0.8 mL/min.

An AIN-93M control diet and an AIN-93M diet supplemented with DMW were prepared by Research Diets, Inc. (New Brunswick, NJ, USA). DMW was added at 4.8% (*v*/*w*). The composition of the diets is shown in Supplemental Table S1.

### 2.4. Animal Care and Experimental Design

The Institutional Animal Care and Use Committee at the University of Florida reviewed and approved the protocol. IL-10^−/−^ C57BL/6 mice and their wild-type counterparts (male, six weeks old) were provided by Jackson Lab (Bar Harbor, ME, USA). They were housed three per cage and acclimated on the AIN-93M control diet for one week. After that, twenty-four IL-10^−/−^ mice were randomly separated into two groups of equal size. One group was given an AIN-93M control diet, while the other group was given the AIN-93M diet supplemented with 4.8% DMW. The same protocol was repeated on the twenty-four WT mice (Table S2). The grouping of mice was not blinded to the researchers. The number of mice in each group was comparable to a published study [16]. Feeding lasted for 154 days (22 weeks). Body weight, food intake, stool consistency, and rectal bleeding were recorded twice a week. Disease activity index (DAI) was calculated based on reduced weight gain, stool consistency, and rectal bleeding, according to a published method with modifications [17]. The reduced weight gain was calculated as the difference between 100% and the ratio of individual mouse weight over the average weight of WT mice on the control diet on the same feeding day. The reduced weight gain was used because mice in this 22-week study had no weight loss, which is typical only in the DSS-induced acute colitis mouse models [17].

For assessment of intestinal permeability on day 142, mice were deprived of food for 6 h before they were gavaged with FITC-dextran (44 mg/100 g weight). After 4 h, blood was sampled from a facial vein and separated into serum. The fluorescence intensity of diluted serum was measured using 485-nm excitation and 585-nm emission.

Mice were euthanized on day 154, and blood was collected by cardiac puncture, and the colon was removed. This study did not control the location of mouse cages and the sequence of measurements.

### 2.5. Quantitative Reverse Transcription PCR (RT-qPCR)

Mouse colon RNA was extracted using RNAzol^®^ RT (Molecular Research Center, Inc., Cincinnati, OH, USA) and reverse-transcribed with iScript cDNA Synthesis kit (Bio-Rad Laboratories, Inc., Hercules, CA, USA). The primers are listed in Supplemental Table S3. The plates containing primers, cDNA, SYBR Green supermix (Bio-Rad Laboratories, Inc., USA), and nuclease-free water were sealed and centrifuged at 3000× *g* for 3 min. Real-Time PCR Detection System (Bio-Rad Laboratories, Inc., Hercules, CA, USA) was used to amplify the genes. GAPDH served as a reference gene, and the relative gene expressions were calculated using the 2^−ΔΔCt^ method.

### 2.6. Analysis of Serum IL-6, IL-1β and TNF-α

Serum IL-6, IL-1β, and TNF-α on day 154 were analyzed using ELISA kits according to the protocols provided by the manufacturers (Invitrogen, Carlsbad, CA, USA).

### 2.7. Gut Microbiome Analysis

Fecal samples from individual mice were collected the day before euthanasia and stored at −80 °C. The bacterial DNA was extracted using the QIAamp Fast DNA Stool Mini Kit (Qiagen, Venlo, The Netherlands). The 16S ribosomal RNA genes (V3-4 region) were amplified. The clean sequences were compared with those in a database (silva-132-99-nb-classifier.qza). Qiime2 scripts written in Linux were used to filter and clean sequences, merge paired-end sequences, and generate taxonomy, rooted-tree, and OTU tables.

### 2.8. Statistical Analysis

Data are expressed as mean ± standard deviation. Parametric data were analyzed with generalized linear regression two-way ANOVA. Non-parametric data (α-diversity and bacterial abundance) were analyzed using aligned rank transform two-way ANOVA. Two-way ANOVA was selected as a statistical method because this study had a two-factor (diet and gene) and two-level design (+ and −). If a significant effect of diet, gene, or diet × gene interaction was detected, post-hoc contrasts were performed for group comparisons. The *p*-values for multiple group comparisons were adjusted using the Benjamini-Hochberg procedure. Analyses were performed in R (Version 4.4.2). PERMANOVA and its pairwise tests (adonis2) were used to assess beta diversity.

## 3. Results and Discussion

### 3.1. DMW Contained Anthocyanins, Ellagic Acid, and Flavonols

The most abundant anthocyanins in DMW were delphinidin-3,5-diglucoside (1008.3 µg/mL) and cyanidin 3,5-diglucoside (1000.5 µg/mL), followed by peonidin 3,5-diglucoside (Table 1). The total content of anthocyanins was 3454.8 µg/mL. DMW also contained a significant amount of ellagic acid (348.9 µg/mL) but a small amount of myricetin (106.1 µg/mL) and quercetin (17.7 µg/mL) (Chromatograms in Supplemental Figure S1). The polyphenol profile of DMW was comparable to a published paper [12]. In this study, the dose of anthocyanins for mice was 16.56 mg/(kg·d). Based on body surface area [18], the human equivalent dose of muscadine wine and anthocyanins was 234 mL and 81 mg, respectively, for a person weighing 60 kg.

### 3.2. DMW Significantly Affected Body Weight but Not DAI

**Figure 1:**
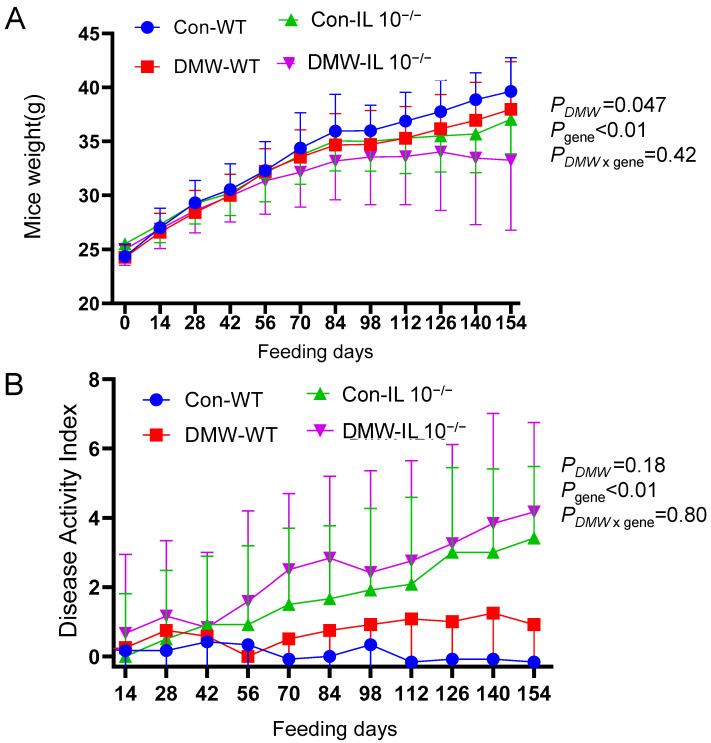
DMW and gene affected body weight (**A**) and Disease Activity Index (**B**) of wild-type (WT) and IL-10^−/−^ mice fed control or DMW-supplemented diet. *n* = 12 mice per group. *p*-Values of two-way ANOVA were shown for day 154.

Both DMW (*p* = 0.047) and gene (*p* < 0.01) significantly affected body weight at day 154 (Figure 1A). DMW had greater impact on the body weight gain in IL-10^−/−^ mice (*p* = 0.052) than in WT mice (*p* = 0.37). IL-10^−/−^ mice had lower body weight than WT mice in the DMW feeding groups (*p* = 0.01), which was consistent with the spontaneous development of colitis in IL-10^−/−^ mice. The impact of DMW on body weight was similar to the result by Kang et al., who found that a diet with 1% Goji Berry for 10 weeks did not alter the body weight of IL-10^−/−^ mice [19].

The symptoms and severity of colitis were assessed using DAI, which is a combined score of reduced weight gain, stool consistency, and rectal bleeding. The maximum score for each of the three symptoms was 4. Therefore, mice with extremely severe colitis receive a maximum DAI of 12. The WT mice showed no sign of colitis throughout the feeding period of 22 weeks. The DAI in IL-10^−/−^ mice gradually increased from zero to about 4.1 after 22 weeks of feeding (Figure 1B). The DAI scores were primarily due to reduced weight gain and soft stool. Severe diarrhea or rectal bleeding was not observed. DAI was significantly affected by gene (*p* < 0.01) but not by diet (*p* = 0.18) or diet x gene interaction (*p* = 0.80). A previous study fed C57BL/6 IL-10^−/−^ mice a control diet for 10 weeks and reported an average DAI of 6.5, which was much higher than the value in this study. The onset and severity of colitis in IL-10^−/−^ mice depend on both the genetic background of the mice and the environmental microbiome. The C57BL/6 IL-10^−/−^ mice typically develop milder and delayed colitis compared to mice of other strains, such as the 29/SvEv or BALB/c mice [10]. It was observed that colitis symptoms can be absent in C57BL/6 IL-10^−/−^ mice in some animal facilities due to the lack of some environmental microbiome [20]. The very mild colitis observed in this study suggested the environmental microbiome in the animal facility did not favor the development of severe colitis. This study did not use antibiotics to accelerate the onset and severity of colitis because antibiotics were known to disrupt the gut microbiome composition [21].

Previous studies using the DSS-induced acute colitis model have shown that anthocyanins reduce DAI in mice [6,22]. DSS-treated mice are characterized by body weight loss of up to 15%, diarrhea, and rectal bleeding after three days. However, these severe symptoms were absent in this study, which may have limited the impact of DMW on DAI. Replacing weight loss with reduced weight gain in the calculation of DAI may also affect its response to diet. Nevertheless, these results suggested that DMW had limited effects on IL-10-deficient mice, an observation shared by a published study that showed the limited effect of dietary curcumin [23]. In vitro studies suggested that the anti-inflammatory activities of curcumin were attenuated in IL-10-deficient mice because part of its full activity depends on IL-10 [23].

### 3.3. DMW Interacted with Gene to Reduce Intestinal Permeability in IL-10^−/−^ Mice

**Figure 2:**
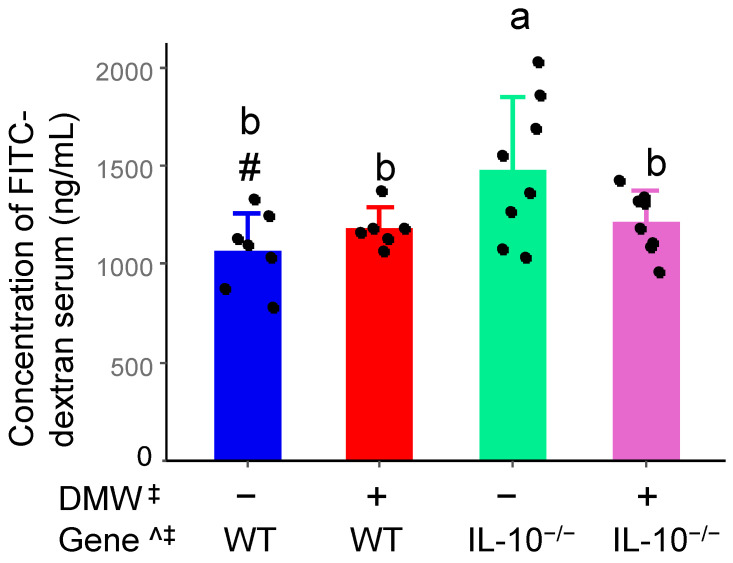
DMW interacted with gene to decrease intestinal permeability (FITC-dextran level on day 142) in IL-10^−/−^ mice (*n* = 6–8). ^ significant effect by gene; ^‡^ significant effect of DMW × gene interaction; ^#^ significant genetic difference in the same dietary group. Data were square-transformed before the analysis. Letters on the columns indicate significant all-pairs-wise differences when there is a significant effect of DMW × gene interaction.

IBD patients have high intestinal permeability, which mainly results from a defective mucosal barrier and exacerbates intestinal inflammation [24]. The intestinal permeability of mice was significantly affected by gene and DMW × gene interaction (Figure 2). DMW interacted with the gene to decrease the intestinal permeability in IL-10^−/−^ mice but did not alter the intestinal permeability in WT mice. The result partially agreed with a published study, which found that grape seed extract for 12 weeks reduced gut permeability in IL-10-deficient mice with mild colitis [25].

Polyphenols have been shown to interact with the presence and absence of the IL-10 gene through multiple mechanisms. For example, epigallocatechin gallate has been found to modulate IL-10 mRNA and protein levels in mice under inflammatory conditions [26]. IL-10 and its receptors regulate immune response mainly through the JAK-STAT signaling pathway [27]. Several polyphenols have been found to directly affect their activities. For instance, hesperidin in combination with cinnamaldehyde significantly decreased p-JAK2 and p-STAT3 and significantly increased the expression of SOCS3 protein in ulcerative colitis [28]. In addition, polyphenols and IL-10 can influence each other’s activities through the gut microbiome because both are known to interact with gut microbiota reciprocally [29]. Since IL-10 is essential for anti-inflammatory signaling and immune homeostasis, its absence is expected to diminish or alter the biological effects of dietary polyphenol interventions such as DMW.

### 3.4. DMW, Gene, and Their Interactions Differentially Affect Gene Expression of Cytokines and Tight Junction Proteins

**Figure 3:**
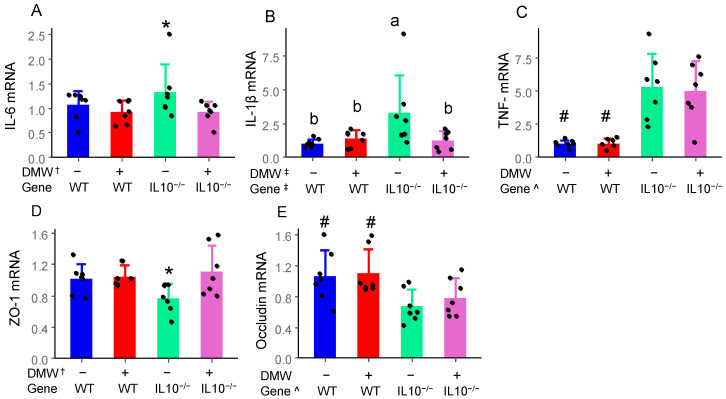
DMW, gene, and their interactions differentially affected relative mRNA levels of IL-6 (**A**), IL-1β (**B**), TNF-α (**C**), ZO-1 (**D**), and occludin (**E**) in the colon of mice (*n* = 7). ^†^ significant effect by DMW; ^ significant effect by gene; ^‡^ significant effect of DMW × gene interaction. * Significant dietary difference in the same genotype. ^#^ significant genetic differences in the same diet group. Letters on the columns indicate significant all-pairs-wise differences when there is a significant effect of DMW × gene interaction.

IL-6, IL-1β, and TNF-α are key pro-inflammatory cytokines involved in the initiation and progression of colitis. DMW and DMW × gene interaction significantly reduced IL-6 and IL-1β mRNA in the colon of IL-10^−/−^ mice, respectively, but not in WT mice (Figure 3A,B). IL-10^−/−^ mice had higher TNF-α than WT mice, but DMW had no impact in either group (Figure 3C). The reduction in mRNA expression of IL-6 and IL-1β in the colon has been consistently observed in mice with induced colitis, including purple-fleshed potatoes, black rice anthocyanin-rich extract, and raspberry anthocyanin-rich fractions [22,30,31]. The lack of DMW’s effect on mRNA level of TNF-α is likely because TNF-α is an early response cytokine regulated via different signaling cascades than IL-6 or IL-1β [32].

The integrity of the epithelial barrier is maintained by tight junction proteins, including ZO-1 and occludin [33]. DMW significantly enhanced the colonic gene expression of ZO-1 in IL-10^−/−^ mice compared to the control diet, but not in WT mice (Figure 3D). The gene expression of occludin in IL-10^−/−^ mice was significantly lower than that in WT mice. However, it was not affected by DMW in either the WT or IL-10^−/−^ mice (Figure 3E). The observation on ZO-1 agreed with a previous study showing that mulberry anthocyanins restored the expression of intestinal tight junction protein ZO-1 [34]. Red wine polyphenols are known to increase mRNA of ZO-1 in HT-29 colon cells [35]. ZO-1 acts as a scaffold protein that connects transmembrane proteins (like claudins and occludins) to the actin cytoskeleton. This helps to form and stabilize tight junctions between cells. The increased ZO-1 mRNA in the colon helps to explain the decreased intestinal permeability in the IL-10^−/−^ mice fed a DMW-supplemented diet.

### 3.5. DMW × Gene Interaction Reduced the Serum TNF-α in IL-10^−/−^ Mice

**Figure 4:**
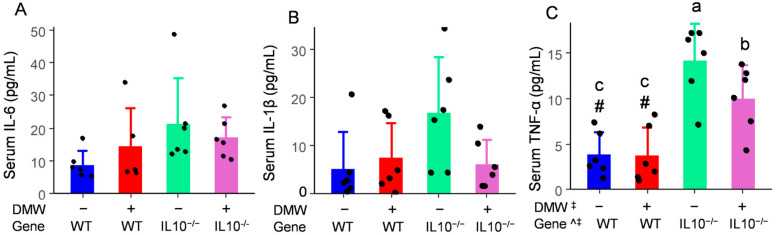
DMW × gene interaction did not affect serum levels of IL-6 (**A**) and IL-1β (**B**), but reduced TNF-α (**C**) in IL-10^−/−^ mice (*n* = 6). ^ significant effect by gene; ^‡^ significant effect of DMW × gene interaction. ^#^ significant genetic differences in the same diet group. Data were square-transformed before the analysis. Letters on the columns indicate significant all-pairs-wise differences when there was a significant effect of DMW × gene interaction.

The mRNA of inflammatory cytokines in the colon reflects a localized early stage of immune response, while the serum levels of IL-6, IL-1β, and TNF-α are indicators of systemic inflammation. This is because serum cytokines are produced by the entire immune system, including those from the colon. Although DMW and DMW × gene interaction affected mRNA of IL-6 and IL-1β in the colon, their serum levels were not impacted (Figure 4A,B), suggesting the localized effect of DMW on IL-6 and IL-1β was stronger in the colon than its systemic activities. Anthocyanins and ellagic acid are known to have extremely low absorption rates, as indicated by their low contents in systemic circulation after oral ingestion. A vast majority of them enter the colon to exert a localized effect [36]. IL-10^−/−^ mice had a higher serum level of TNF-α than WT mice due to genetic impact (Figure 4C)**,** which was consistent with TNF-α mRNA in the colon. Although DMW alone did not affect serum TNF-α (*p* = 0.06), DMW × gene interaction significantly decreased the serum content of TNF-α in IL-10^−/−^ mice but not in WT mice. Serum TNF-α appeared to be more responsive to dietary DMW, likely due to its role as a master cytokine in the pathogenesis of IBD and its status as a primary target for therapeutic intervention. The systemic anti-inflammatory activity of DMW was consistent with previous research, which reported that black rice extract and blackcurrant reduced serum levels of IL-1β, TNF-α, and IL-6 in mice with DSS-induced colitis [37,38].

### 3.6. DMW Did Not Affect α-Diversity but Altered the Beta-Diversity of the Gut Microbiome

**Figure 5:**
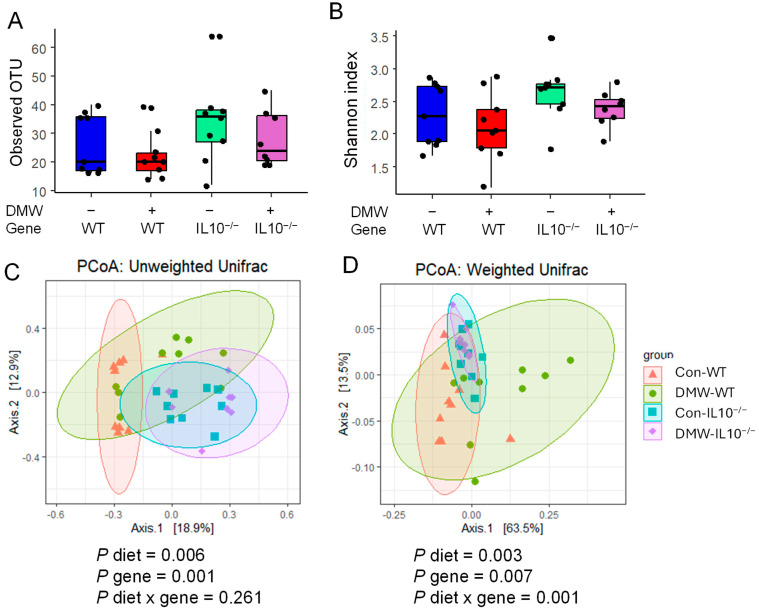
DMW or gene did not change the α-diversity of gut microbiome measured by observed OTU (**A**) or Shannon index (**B**); both DMW and gene altered bacterial β-diversity based on PCoA plots of unweighted UniFrac distance (**C**) and weighted UniFrac distance (**D**) (*n* = 8–9). The *p*-values for β-diversity were from PERMANOVA tests.

DMW or gene did not affect α-diversity among all four groups (Figure 5A,B). Observed OTU is calculated based on the richness of species, and Shannon is a balanced index based on both the richness and evenness of species. A similar study also found that 8 weeks of pomegranate extract did not affect the Shannon index of the cecum of IL-10^−/−^ mice [39]. IL-10^−/−^ mice in this study developed very mild colitis after 22 weeks of feeding, most likely due to an unfavorable environmental microbiome. This may have blunted the impact of the gene on α-diversity.

Both diet and gene significantly affected the β-diversity of gut microbiomes based on the PCoA plot of unweighted and weighted UniFrac distances and PERMANOVA tests (Figure 5C,D). Significant diet x gene interaction was only observed in weighted UniFrac distances, but not in unweighted UniFrac distances. Pairwise comparison showed that DMW significantly altered β-diversity in the WT mice but not in the IL-10^−/−^ mice (Tables S4 and S5). These results agreed with a similar study that reported that pomegranate extract did not alter beta-diversity in IL-10^−/−^ mice [39]. Both unweighted and weighted UniFrac are phylogenetic methods of comparing microbial communities. Unweighted UniFrac detects compositional change according to the presence and absence of taxa, while the weighted UniFrac detects structural change according to the relative abundance of taxa. Unweighted UniFrac is more sensitive to rare taxa than weighted UniFrac distance [40]. The altered β-diversity in IL-10^−/−^ mice compared to WT mice may have reduced the gut microbiome’s responsiveness to DMW in the diet.

### 3.7. DMW and DMW × Gene Interaction Altered the Relative Abundance of Some Gut Microbes

**Figure 6:**
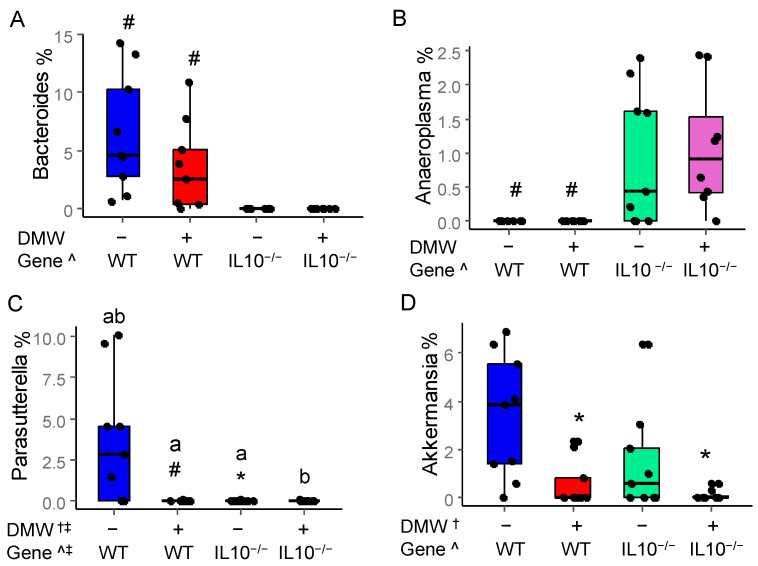
DMW, gene, and their interactions altered the relative abundance of gut microbiome at the genus level for (**A**) *Bacteroides*, (**B**) *Anaeroplasma*, (**C**) *Parasutterella*, and (**D**) *Akkermansia*. ^†^ significant effect by DMW; ^ significant effect by genotype; ^‡^ significant effect of DMW × gene interaction; * significant dietary difference in the same genotype; ^#^ significant genetic difference in the same diet group. Letters on the columns indicate significant all-pairs-wise differences when there is a significant effect of DMW × gene interaction.

The relative abundance of four gut microbes at the genus level is depicted in Figure 6. DMW did not affect the abundance of *Bacteroides* and *Anaeroplasma*, whereas only the gene had significant impacts (Figure 6A,B). WT mice had a higher abundance of *Bacteroides* than IL-10^−/−^ mice, while the opposite was observed for *Anaeroplasma. Bacteroides* are known to modulate immune function and ferment complex polysaccharides to produce SCFA [41], which was the possible reason for their higher relative abundance in WT mice. *Anaeroplasma* was an opportunistic pathogen which has been reported to induce diverse immune responses in human diseases such as colon cancer [42], which was the possible reason that there was a higher relative abundance of them in IL-10^−/−^ mice than in WT mice.

The abundance of *Parasutterella* was affected by DMW, gene, and their interaction. *Parasutterella* was a potential pathogen because they are involved in bile acid metabolism and are associated with inflammatory bowel diseases [43]. DMW significantly reduced the abundance of *Parasutterella* in IL-10^−/−^ mice (Figure 6C), which may provide benefits to alleviate colitis.

DMW reduced the relative abundance of *Akkermansia* in both WT and IL-10^−/−^ mice. IL-10^−/−^ mice had a lower abundance of *Akkermansia* than the WT mice (Figure 6D). Previous studies also found a lower abundance of *Akkermansia* in IL-10^−/−^ mice and suggested that *Akkermansia* correlates negatively with gut inflammation [44,45]. *Akkermansia* are mucin-degrading bacteria in the phylum of *Verrucomicrobia* colonizing the intestinal mucosa of humans and rodents. An optimal level of *Akkermansia* is critical for gut health because a lower abundance may impair mucus renewal and compromise the gut barrier [46]. However, a higher abundance can lead to over-degradation and thinning of the mucus layer. The activity of DMW in IL10^−/−^ mice appeared to be similar to NLRP6 [44], which was shown to protect IL10^−/−^ mice from colitis by limiting colonization of *Akkermansia*.

### 3.8. Spearman Correlation Between mRNA and Significantly Changed Gut Microbiota

**Figure 7:**
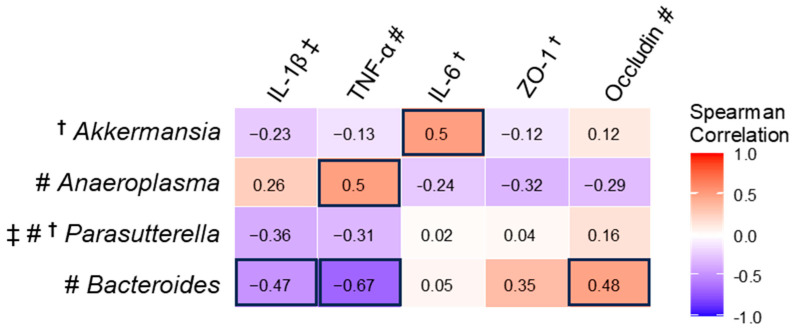
Gut microbiome at the genus level correlated with relative mRNA levels of cytokines and tight junction proteins in the colon. ^†^ significantly affected by DMW; ^#^ significantly affected by gene; ^‡^ significantly affected by DMW × gene interaction. Rectangles with dark outlines indicate significant correlations. Numbers in the rectangles are the correlation coefficients.

*Bacteroides* negatively correlated with colon IL-1β and TNF-α mRNA, which was consistent with its lower abundance in IL-10^−/−^ mice compared to WT mice, in contrast to higher colon mRNA of IL-1β and TNF-α in IL-10^−/−^ mice (Figure 7). *Bacteroides* correlated positively with occludin because both had a lower amount in IL10^−/−^ mice than in WT mice. *Bacteroides* isolates were found to inhibit the IL-1β-triggered inflammatory response in CFTR^−/−^ Caco-2 intestinal epithelial cells [47]. Another study found that the administration of *Bacteroides fragilis* inhibited the gene expression of inflammatory cytokines (TNF-α, IL-1β, IL-6) but enhanced the gene expression of tight junction proteins (ZO-1, occludin, claudin-1) [48]. This suggests that *Bacteroides* may be a potential probiotic for treating colitis. *Anaeroplasma* positively correlated with TNF-α because both had higher levels in IL10^−/−^ Mice than in WT mice, suggesting *Anaeroplasma* may have contributed to immune activation. *Akkermansia* positively correlated with IL-6. The impact of *Akkermansia* on inflammatory cytokine IL-6 depends on the biological context. *Akkermansia* was found to increase transcriptional levels of IL-6 in the colon in mice treated with Azoxymethane/DSS and antibiotics [49], whereas IL-6 was reduced by *Akkermansia* in mice with DSS-induced colitis [50].

The dose of DMW used in this study was equivalent to 1.5 glasses of wine for a 60-kg adult, which is twice the daily amount estimated for regular wine drinkers (0.5–0.7 glasses of wine per day). However, the obstacles of high dose and alcohol content can be solved by non-alcoholic wines, which have gained popularity among consumers in recent years. Such a trend will help to translate these preclinical results to the human population.

The IL-10^−/−^ C57BL/6 mice in this study developed very mild colitis after 22 weeks of feeding, which may have limited the bioactivities of DMW; this remains a limitation of this study. The 16S rRNA sequencing resolved the gut microbiome to the genus level. The lack of abundance at the species level or functional data was another limitation of this study. Other limitations included not considering the cage effect for β-diversity analysis, lack of histological data, and possible genetic compensation for the lost IL-10 function in the knockout mice. It should also be noted that the significant statistical effects of DMW × gene interaction should not be misunderstood as biological signaling.

## 4. Conclusions

This study was among the few that investigated how chronic intake of polyphenols impacts colitis development in genetically susceptible mice compared to wild-type counterparts. Results showed that DMW and DMW × gene interaction reduced intestinal permeability, gene expression of inflammatory cytokines, and serum TNF-α, and enhanced the gene expression of tight junction proteins (ZO-1) in IL-10^−/−^ mice. DMW significantly reduced the relative abundances of *Parasutterella* and *Akkermansia* in the IL-10^−/−^ mice. These results suggested that polyphenols from DMW moderately alleviated the development of colitis in IL-10^−/−^ mice and could be a useful dietary strategy for IBD prevention. Results in this study should be verified in a future study using mouse strains that are more susceptible to colitis development, such as the 29/SvEv or BALB/c mice. Modulation of the gut microbiome should be verified using whole-genome sequencing.

## Figures and Tables

**Table 1:** Contents of polyphenols in DMW.

Polyphenols	Concentration (µg/mL)
Delphinidin-3,5-diglucoside	1008.3 ± 62.7
Cyanidin 3,5-diglucoside	1000.5 ± 62.2
Petunidin 3,5-diglucoside	562.3 ± 36.7
Pelargonidin 3,5-diglucoside	49.9 ± 1.8
Peonidin 3,5-diglucoside	590.3 ± 39.6
Malvidin 3,5-diglucoside	243.5 ± 15.5
Total anthocyanins	3454.8 ± 217.9
Myricetin	106.1 ± 18.3
Quercetin	17.7 ± 2.5
Kaempeferol	3.7 ± 0.7
Ellagic acid	348.9 ± 16.9
Total other polyphenols	476.4 ± 36.5

Data are mean ± SD of triplicate tests.

## Data Availability

The original contributions presented in the study are included in the article/Supplementary Material; further inquiries can be directed to the corresponding author.

## Supplementary Materials

The following supporting information can be downloaded at: https://www.mdpi.com/article/10.3390/nu17142327/s1, Table S1. Nutrition composition of the control diet, DMW, and diet with 4.8% (*w*/*v*) DMW. Table S2: Experimental design and mouse grouping. Table S3. Genes and primers for RT-qPCR. Table S4 Pairwise-PERMANOVA results based on unweighted UniFrac distance. Table S5 Pairwise-PERMANOVA results based on weighted UniFrac distance. Figure S1: HPLC chromatogram of anthocyanins (A) and other polyphenols (B) in DMW using 520 nm and 360 nm detection, respectively. Peaks 1, 2, 3, 4, 5 and 6 in panel A are delphinidin-3,5-diglucoside, cyanidin-3,5-diglucoside, petunidin-3,5-diglucoside, pelargonidin-3,5-diglucoside, peonidin-3,5-diglucoside and malvidin-3,5-diglucoside, respectively. Peaks 7, 8, 9 and 10 in panel B are ellagic acid, myricetin, quercetin, and kaempferol, respectively.

## Abbreviations

DAI	Disease activity index
DMW	Dealcoholized muscadine wine
DSS	Dextran sulfate sodium
FITC	Fluorescein isothiocyanate
IBD	Inflammatory bowel diseases
WT	Wild type
