# Supplementary material for: Effect of Dealcoholized Muscadine Wine on the Development of Spontaneous Colitis and Gut Microbiome in IL-10−/− Mice
Source: Nutrients. 2025 Jul 16;17(14):2327. doi: 10.3390/nu17142327 (PMC12298891; doi:10.3390/nu17142327)
Supplement: Supplementary file 1 [file nutrients-17-02327-s001.zip › nutrients-3702932-supplementary.pdf]

Table S1. Nutrition composition of the control diet, DMW, and diet with 4.8% (w/v) DMW

Ingredients	Control diet (w/g)	DMW (w/g)	Diet with 4.8% DMW (w/g)
Casein	140	0.275	139.725
L-Cystine	1.8	0	1.8
Corn Starch	450	0	450
Maltodextrin10	125	0	125
Fructose	24.3	0.163	24.137
Dextrose	21.7	0.284	21.416
Sucrose	100	0.014	99.986
Cellulose	50	0.467	49.533
Soybean oil	40	0	40
tBHQ	0.008	0	0.008
Mineral Mix S10022M	35	0.34	34.66
Vitamin Mix V10037	10	0	10
Choline Bitartrate	2.5	0	2.5
DMW/ml		48	51.84
FD&C Red Dye #40			0.05
Total wet weight/g			1050.654
Total dry weight/g	1000.308		1006.74

Table S2: Experimental design and mouse grouping

Mice	7-day acclimation diet	22-week feeding diet
WT C57BL/6 mice	AIN-93M diet	AIN-93M diet
WT C57BL/6 mice	AIN-93M diet	AIN-93M diet supplemented with 4.8% DMW
IL-10$^{-/-}$ C57BL/6 mice	AIN-93M diet	AIN-93M diet
IL-10$^{-/-}$ C57BL/6 mice	AIN-93M diet	AIN-93M diet supplemented with 4.8% DMW

Table S3. Genes and primers for RT-qPCR

NCBI ID#	Sequence Name	Forward primer	Reverse primer
IL-6	NM_031168.2	TGATGGATGCTACCAAACTGGA	TCTCTCTGAAGGACTCTGGCTT
IL-1β	NM_008361.4	TGCCACCTTTTGACAGTGATGA	CATCAGGACAGCCCAGGTCAA
TNF-α	NM_013693.3	CTGAACTTCGGGGTGATCGGT	GTTTGCTACGACGTGGGCTA
ZO-1	NM_009386.2	CGAGATGCTGGGACTGACCA	GACGATCAACCGCATTTGGC
Occludin	NM_008756.2	AGTGAAGAGTACATGGCTGCTG	CGTCTGTCATAATCTCCCACCA
GAPDH	NM_008084.3	GGTGAAGGTCGGTGTGAACG	AATGAAGGGGTCGTTGATGGC

Table S4 Pairwise-PERMANOVA results based on unweighted UniFrac distance

Groups	Permutation	F	p-value	p.adj.fdr
Con-WT_***vs***_DMW-WT	999	2.6769	0.005	0.006
Con-WT_***vs***_Con-IL-10	999	3.6888	0.001	0.003
Con-WT_***vs***_DMW-IL-10	999	4.9967	0.001	0.003
DMW-WT_***vs***_Con-IL-10	999	2.5963	0.002	0.004
DMW-WT_***vs***_DMW-IL-10	999	2.7378	0.003	0.004
Con-IL-10_***vs***_DMW-IL-10	999	0.9754	0.449	0.449

Table S5 Pairwise-PERMANOVA results based on weighted UniFrac distance

Groups	Permutation	F	p-value	p.adj.fdr
Con-WT_*vs*_DMW-WT	999	7.4695	0.010	0.015
Con-WT_vs_Con-IL-10	999	5.5282	0.001	0.006
Con-WT_vs_DMW-IL-10	999	5.5861	0.002	0.006
DMW-WT_vs_Con-IL-10	999	4.7593	0.020	0.024
DMW-WT_vs_DMW-IL-10	999	5.7565	0.005	0.010
Con-IL-10_vs_DMW-IL-10	999	1.1571	0.290	0.290

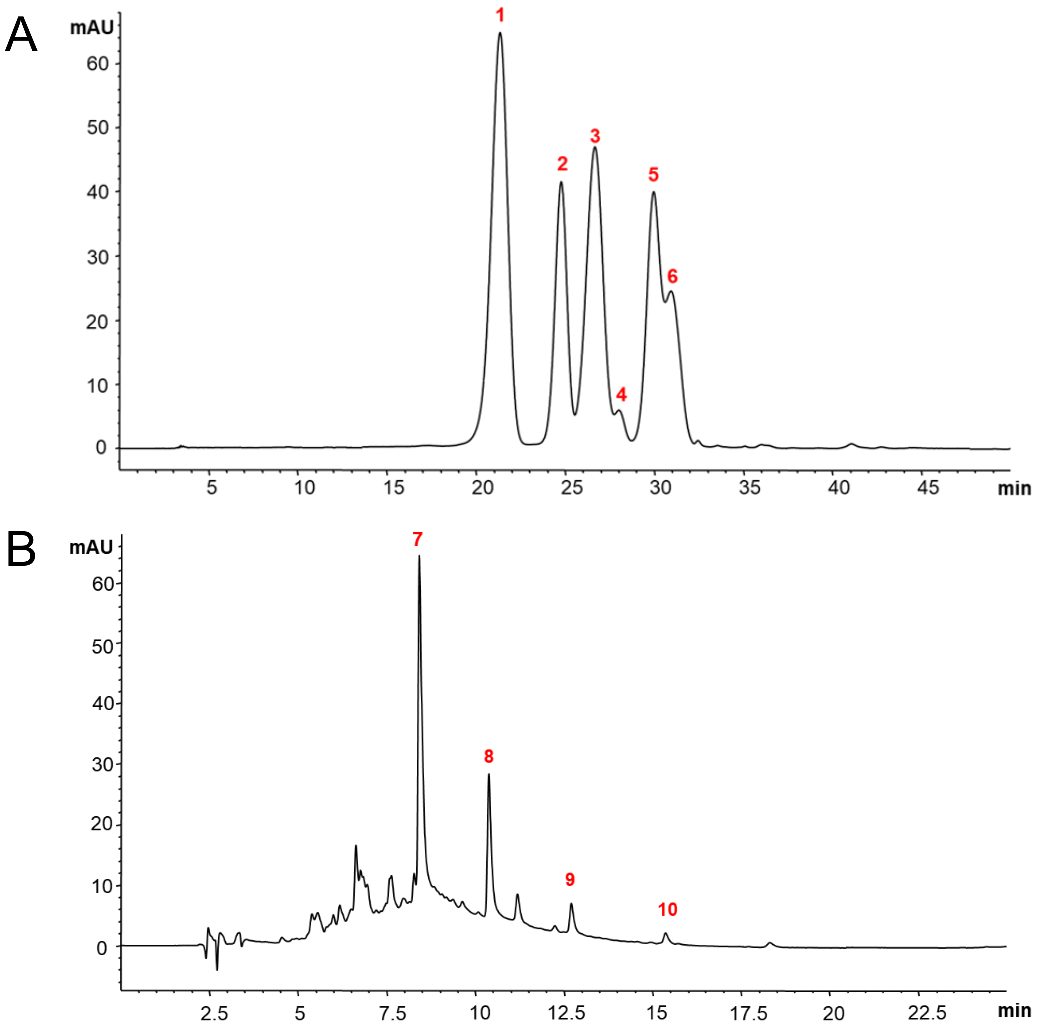

Figure S1: HPLC chromatogram of anthocyanins (A) and other polyphenols (B) in DMW using 520 nm and 360 nm detection, respectively. Peaks 1, 2, 3, 4, 5 and 6 in panel A are delphinidin-3,5-diglucoside, cyanidin-3,5-diglucoside, petunidin-3,5-diglucoside, pelargonidin-3,5-diglucoside, peonidin-3,5-diglucoside and malvidin-3,5-diglucoside, respectively. Peaks 7, 8, 9 and 10 in panel B are ellagic acid, myricetin, quercetin, and kaempferol, respectively.
